# PDE4 inhibitors: potential protective effects in inflammation and vascular diseases

**DOI:** 10.3389/fphar.2024.1407871

**Published:** 2024-06-10

**Authors:** Tianfei Fan, Wenjing Wang, Yao Wang, Mingtang Zeng, Yi Liu, Shuyao Zhu, Lin Yang

**Affiliations:** ^1^ Department of Pharmacy, West China Hospital, Sichuan University, Chengdu, China; ^2^ Department of Pharmacy, Institute of Metabolic Diseases and Pharmacotherapy, West China Hospital, Sichuan University, Chengdu, China

**Keywords:** phosphodiesterase 4, inhibitor, inflammation, vascular disease, treatment

## Abstract

Phosphodiesterase 4 (PDE4) inhibitors are effective therapeutic agents for various inflammatory diseases. Roflumilast, apremilast, and crisaborole have been developed and approved for the treatment of chronic obstructive pulmonary disease psoriatic arthritis, and atopic dermatitis. Inflammation underlies many vascular diseases, yet the role of PDE4 inhibitors in these diseases remains inadequately explored. This review elucidates the clinical applications and anti-inflammatory mechanisms of PDE4 inhibitors, as well as their potential protective effects on vascular diseases. Additionally, strategies to mitigate the adverse reactions of PDE4 inhibitors are discussed. This article emphasizes the need for further exploration of the therapeutic potential and clinical applications of PDE4 inhibitors in vascular diseases.

## 1 Introduction

Phosphodiesterase 4 (PDE4) is primarily present in immune cells, epithelial cells, and brain cells, acting as an intracellular non-receptor enzyme that regulates inflammation and epithelial integrity. Inhibiting PDE4 increases cyclic adenosine monophosphate (cAMP) levels, thereby regulating various genes and proteins to exert multiple effects and functions. Currently, PDE4 is regarded as a promising and effective therapeutic target for pulmonary diseases, skin diseases, and neurological disorders. Over the past few decades, numerous PDE4 inhibitors have been designed and synthesized. Roflumilast, apremilast, and crisaborole are approved for treating inflammatory airway diseases, psoriatic arthritis, and atopic dermatitis, respectively. However, the efficacy of these drugs is often accompanied by adverse reactions, including nausea, vomiting, and gastrointestinal disturbances. With the continuous development of drugs, PDE4 inhibitor have partially mitigated adverse reactions and achieved improved efficacy. It is well established that PDE4 inhibitors play a crucial role in inflammation. Inflammation underlies the pathogenesis of many vascular diseases. However, the mechanism of PDE4 inhibitors in vascular diseases remains unclear. This review summarizes the roles of PDE4 inhibitors in inflammation and vascular diseases, and discusses prospects for mitigating the adverse reactions of these inhibitors.

## 2 The PDE4 subfamily

Phosphodiesterases (PDEs) comprise 11 subfamilies (PDE1-PDE11), responsible for degrading cyclic adenosine monophosphate (cAMP) and cyclic guanosine monophosphate (cGMP) (Zuccarello et al., 2023). cAMP and cGMP are second messengers that regulate numerous cellular pathways. They open cyclic nucleotide-gated ion channels and stimulate protein kinases (PKA and PKG) activated by cAMP or cGMP (Gutierrez-Rodelo et al., 2023). Downstream targets of PKA and PKG comprise receptors, ion channels, cytoskeletal proteins, and transcription factors. These components regulate various functions such as neuronal excitability, cellular metabolism, and gene expression. Therefore, PDEs play a critical role in cellular function.

The PDE4 subfamily, including four isoforms (PDE4A, PDE4B, PDE4C, and PDE4D), is the largest among the 11 PDE families. PDE4 selectively degrades cAMP and is widely distributed in various tissues and cells of the human body. These tissues and cells include the brain, kidneys, cardiac myocytes, endothelial cells, and immune cells. PDE4 is involved in pathophysiological processes, including monocyte and macrophage activation, neutrophil infiltration, vascular smooth muscle proliferation, vasodilation, and myocardial contraction. These show PDE4 impacts the central nervous system, cardiovascular function, and immune-inflammatory systems.

The distribution of PDE4A, PDE4B, PDE4C, and PDE4D in the brain and peripheral tissues is determined by Viktor Lakics et al. Their findings reveals that PDE4A expression is higher in the brain compared to peripheral tissues. However, in peripheral tissues, PDE4A exhibits the highest content in muscles, thyroid, stomach, lungs, and spleen. PDE4B is the most strongly expressed isoform of PDE4, with the highest expression in the brain, followed by spleen, lungs, bladder, thyroid, muscles, and heart. PDE4C is detected in the spleen, lungs, stomach, heart, and brain, but expression is always minimal. PDE4D is highest in bladder and muscle tissues, followed by thyroid, heart, kidneys, lungs, and stomach (Lakics et al., 2010). Understanding the distribution of PDE4 in the human body is essential for identifying potential targets of PDE4 inhibitors in different diseases (Goonathilake et al., 2022).

## 3 Clinical applications of PDE4 inhibitors

Targeted inhibiting PDE4 has been demonstrated as an effective therapeutic strategy for treating diseases such as asthma, chronic obstructive pulmonary disease (COPD), psoriasis, atopic dermatitis (AD), inflammatory bowel disease (IBD), rheumatoid arthritis (RA), lupus, neuropathy, depression, memory enhancement, and emesis (Schick and Schlegel, 2022). Nonetheless, the clinical application of PDE4 inhibitors has been consistently hindered by their adverse reactions.

Rolipram, a first-generation PDE4 inhibitor, was initially discovered by Schering AG in the early 1990s. It is considered to have potential in treating neurological disorders such as depression and cognitive dysfunction. Despite its potential pharmacological effects, it has a narrow therapeutic window. Adverse reactions frequently occur during clinical trials, such as nausea, vomiting, and headache (Crocetti et al., 2022). The adverse reactions of rolipram have restricted its clinical use.

Roflumilast, the second-generation PDE4 inhibitor, became the first marketed PDE4 inhibitor. It gained approval for treating severe COPD and asthma in 2010 and 2011, respectively. Although roflumilast demonstrates improved performance in clinical trials compared to rolipram, gastrointestinal adverse reactions still occur 9.5% of cases, manifesting as diarrhea, nausea, headache, weight loss, urinary tract infections, and psychiatric disorders (Asfaw, 2020). Considering the relative equilibrium between efficacy and safety, along with proper dosing strategy and comprehensive assessment of drug safety, roflumilast undeniably confers greater benefits than risks to patients.

Apremilast, the third-generation PDE4 inhibitor, received approval in 2014 for treating psoriatic arthritis (PsA) and moderate to severe plaque psoriasis in adults. Nonetheless, patients encounter adverse reactions, such as headache (5.9%), abdominal pain (2%), depression (1%), weight loss (10%), nausea (8.9%), diarrhea (7.7%), vomiting (3.2%), nasopharyngitis (2.6%), and upper respiratory tract infections (3.9%) (Gooderham and Papp, 2015). Moreover, further clinical data are necessary to sufficiently demonstrate its therapeutic efficacy in children and adolescents with moderate to severe psoriasis (Paller et al., 2020). Apart from PsA and psoriasis, apremilast is utilized for treating various inflammatory diseases such as IBD, Behcet’s syndrome (BS), ankylosing spondylitis (AS), RA, frontal fibrosing alopecia, atopic dermatitis (AD), and discoid lupus erythematosus. Moreover, it is currently undergoing clinical trials (Honma and Hayashi, 2021).

Crisaborole was approved for topical treatment of atopic dermatitis (AD) in 2016 (Kailas, 2017). Local treatment with crisaborole does not cause gastrointestinal adverse reactions compared to systemic treatment. Pharmacokinetic studies have shown that crisaborole, when applied locally, is rapidly absorbed and metabolized into two main inactive metabolites (AN-7602 and AN-8323), thereby reducing the risk of systemic adverse reactions (Zane et al., 2016). Although current study indicates that 2% crisaborole is a safe and effective medication for patients with atopic dermatitis (AD), its longterm efficacy and safety for AD patients under 2 years old remain unclear. Additionally, there is insufficient evidence to prove its safety or efficacy superiority over other existing topical treatment medications. Overall, these drugs represent breakthroughs and achievements in PDE4 inhibitors.

## 4 Anti-inflammatory effects of PDE4 inhibitors

Our understanding of PDE4 function comes primarily from experimental studies involving PDE4 inhibitors. These small-molecule compounds, such as the first-generation inhibitor rolipram, and the second-generation inhibitor roflumilast and cilomilast, have shown extensive pharmacological effects *in vitro* and *in vivo*. These include anti-inflammatory and immunomodulatory effects (Kim et al., 2021), antidepressant and antipsychotic effects (Martinez et al., 2021), and cognitive enhancement (Wei et al., 2023). These findings clearly demonstrate the critical functions of PDE4 in cells and physiology. Among them, PDE4 has been studied most extensively in inflammation. In fact, PDE4 is the major subtype of PDE enzyme expressed in immune and inflammatory cells. Studies have shown that inhibiting PDE4 effectively suppresses various inflammatory responses *in vitro* and *in vivo* (Xu et al., 2020). More importantly, many PDE4 inhibitors under development are effective in animal models of inflammatory diseases, including asthma, COPD, psoriasis, IBD, and rheumatoid arthritis (Peng et al., 2020).

### 4.1 Anti-inflammatory effects of rolipram

In pneumococcal pneumonia mice, rolipram inhibits lung injury. It reduces the levels of the proinflammatory cytokines (tumor necrosis factor α (TNF-α) and interleukin-6 (IL-6)) by increasing proresolving protein annexin A1 (AnxA1) expression (Tavares et al., 2016). Furthermore, rolipram suppresses inflammation and sepsis induced by *Escherichia coli* though inhibiting the MAP kinase and nuclear factor κB (NF-κB) signaling pathway. It increases the production of anti-inflammatory factor (IL-10), and reduces the production of pro-inflammatory factors (IL-1β, IL-5, IL-6, IL-12, TNF-α) (Lu et al., 2020). Additionally, a study has shown that rolipram improves endotoxin-induced cardiac dysfunction by upregulating the expression of dual specificity phosphatase 1 (DUSP1), which suppresses the secretion of TNF-α and IL-6 (Ji et al., 2020). The report by Maier C et al. has demonstrated that rolipram and apremilast decrease the differentiation of M2 macrophages, and reduce skin fibrosis by interfering with the release of IL-6 from macrophages (Maier et al., 2017).

### 4.2 Anti-inflammatory effects of roflumilast

Roflumilast is still considered an effective anti-inflammatory drug for regulating airway inflammation (Kawamatawong, 2017). It induces heme oxygenase-1 (HO-1) expression and inhibits NF-κB, p38 mitogen-activated protein kinases (MAPK), and JNK activation, thereby suppressing the production of TNF-α and inflammation in macrophages (Kwak et al., 2005). Meanwhile, the results from ovalbumin (OVA)-induced airway inflammation model in guinea pigs suggest that roflumilast reduces specific airway resistance. It decreases numbers of circulating leukocytes and eosinophils, which associated with reducing the concentrations of IL-4, IL-5 and TNF-α (Urbanova et al., 2017). It is well known that irreversible or partially reversible airway obstruction in asthma is associated with airway remodeling. Roflumilast in OVA-induced asthmatic mice inhibits airway inflammation, airway remodeling, airway hyperreactivity (AHR). It reduces the levels of IL-4, IL-5 and IL-13 from Th2 cells in bronchoalveolar lavage fluid, which may be related to the stem cell factor (SCF)/c-kit pathway (Kim et al., 2016). Additionally, roflumilast improves bladder dysfunction in diabetic rats, and decreases inflammation and the expression of TNF-α, IL-6 and IL-1β in detrusor smooth muscle (Ding et al., 2019).

### 4.3 Anti-inflammatory effects of apremilast

In preclinical models of psoriasis and arthritis, apremilast reduces the epidermal thickness of lesional skin. It suppresses the abnormal proliferation and expression of TNF-α, IL-12, IL-23 and ICAM-1 (Schafer et al., 2010). Additionally, within 10 days after arthritis onset, apremilast significantly inhibits spontaneous release of TNF-α, and reduces the severity of arthritis in mice without apparent side effects (McCann et al., 2010). Apremilast alleviates murine ulcerative colitis by modulating mucosal immunity though inhibiting the secretion of TNF-α, IFN-γ, IL-1β, IL-2, and IL-6. It also activates PKA-CREB and Epac-Rap1 pathways and subsequently suppressed MAPK, NF-κB, PI3K-mTOR, and JAK-STAT-SOCS3 activation (Li et al., 2019). Furthermore, apremilast suppresses pulmonary inflammation and acute lung injury by reducing myeloperoxidase activity, TNF-α levels, and the infiltration of alveolar cells (Imam et al., 2019). Apremilast inhibits the expression and secretion of pro-inflammatory cytokines, chemokines, and adhesion molecules, including granulocyte-macrophage colony-stimulating factor (GM-CSF), CXC motif chemokine ligand 10 (CXCL10), chemokine (C-C motif) ligand 2 (CCL2), vascular cell adhesion molecule 1 (VCAM-1), E-selectin, and matrix metalloproteinase-9 (MMP9), in TNF-α-induced human umbilical vein endothelial cells (HUVEC) (Otto et al., 2022).

### 4.4 Anti-inflammatory effects of ibudilast

Ibudilast, a well-tolerated oral PDE4 inhibitor, is currently used to treat asthma and stroke. Ibudilast reduces TNF and IL-12 expression in synovial fibroblasts of rheumatoid arthritis. It also inhibits Th17 cell responses and exhibits immunomodulatory activity in experimental arthritis (Clanchy and Williams, 2019). Moreover, ibudilast alleviates acute respiratory distress syndrome in neonatal mice by reducing inflammatory factors (TNF-α, IL-1ß, IL-6 and monocyte chemotactic protein-1 (MCP-1)) and inhibiting apoptosis (Yang et al., 2020). Additionally, ibudilast mitigates Alzheimer’s disease by targeting inflammation and Toll-like receptor signaling and the ubiquitin/proteasome pathway. Thus, it improves hippocampal-dependent spatial memory deficits and microgliosis (Oliveros et al., 2023).

### 4.5 Anti-inflammatory effects of cilomilast

The report by Xu Man et al. demonstrates that cilomilast improves renal dysfunction in cisplatin-induced acute kidney injury. Cilomilast inhibits inflammation by reducing the expression of IL-6, IL-1β, TNF-α and MCP-1, which associated with sirtuin 1, PI3K, and phosphorylated AKT (Xu et al., 2020). Cilomilast suppresses IL-1β and TNF-α, and neutrophilic inflammation, thereby alleviating acute lung injury induced by lipopolysaccharide in mice (Liu et al., 2018).

In conclusion, this data strongly supports the effectiveness and promise of PDE4 as a drug target for various inflammatory diseases.

## 5 Effects of PDE4 inhibitors on vascular

Inflammation serves as the foundation for many diseases, particularly cardiovascular diseases. Currently, our understanding of cAMP signaling almost originates from its role in the cardiovascular system. PDE4, a key hydrolyzing enzyme of cAMP, plays a pivotal role (Colombe and Pidoux, 2021). Extensive study has shown that PDE4 influences numerous complex signaling pathways that regulate cardiac functions (Kamel et al., 2023). In cardiomyocytes, cAMP activates PKA, phosphorylates myofilament proteins, drives β-adrenergic, and enhances cardiac function (Rampersad et al., 2010). Moreover, PDE4 exerts varying effects on large vascular, small vascular, microvascular, and cerebral vascular.

### 5.1 PDE4 inhibitors delay large vascular remodeling

Rolipram inhibits smooth muscle cell apoptosis through the cAMP-PKA-pBad axis, thereby improving vascular remodeling and attenuating abdominal aorta aneurysm formation induced by Ang II in mice (Gao et al., 2022). In a mouse model of femoral artery endothelial injury, rolipram significantly decreases platelet-mediated neutrophil recruitment at the site of vascular injury. It is primarily mediated by downregulation of P-selectin-induced activation of Mac-1 (Totani et al., 2014). Furthermore, roflumilast reduces neointimal formation after femoral artery vascular injury, which involves the Epac-dependent manner by inhibiting the expression of VCAM-1 and histone methylation (Lehrke et al., 2015). Izikki M et al. found that roflumilast reduces IL-6 and MCP-1 expression, inhibits cell proliferation, and alleviates pulmonary vascular remodeling and pulmonary hypertension induced by chronic hypoxia or monocrotaline (Izikki et al., 2009). In addition, apremilast has the protective effects in atherosclerosis via SIRT1 by reducing scavenger receptors, LOX-1 and CD36 levels (Sui and Yu, 2022).

### 5.2 PDE4 inhibitors promote vasodilation of small and medium vascular

Rolipram increases retinal vascular diameter in a dose-dependent manner, leading to retinal vasodilation, with no significant effects on systemic blood pressure, heart rate, or retinal blood flow (Miwa et al., 2009). Moreover, rolipram alleviates Ang II-induced smooth muscle cell contraction and hypertension in mice by activating the PKA-AMPK signaling pathway and inhibiting MYPT1-MLC phosphorylation (Fan et al., 2022). Apremilast alleviates vascular leakage and inflammatory cell infiltration in the retina by regulating Th17 and Treg through the PI3K/AKT/FoxO1 pathway, thereby improving autoimmune uveitis (Chen et al., 2020). Results from Cheng Dongmei et al. indicate that the hypotensive effect of RO 20-1724 (a selective PDE4 inhibitor) on hypertensive rats is mediated by renal vascular smooth muscle cells rather than endothelial cells (Cheng et al., 2010). Ro-20-1724 also attenuates relaxant response to β-adrenergic stimulation and vascular tone in mesenteric arteries from rats with heart failure (Wang et al., 2023).

### 5.3 PDE4 inhibitors improve microvascular circulation

Rolipram reduces microvascular complications, microcirculatory disturbances, capillary leakage, renal injuries, and pulmonary injuries in a rodent animal model of *in vitro* resuscitation (Wollborn et al., 2019). Additionally, rolipram effectively regulates the total cAMP hydrolytic activity in pulmonary microvascular endothelial cells, which is modulated by the intracellular cAMP content via both post-translational and synthetic mechanisms (Zhu et al., 2004). In sepsis and systemic inflammation, rolipram or roflumilast increase endothelial cAMP levels, leading to stabilize the endothelial barrier, increase microcirculatory flow in mesenteric venules and block capillary leakage (Schick et al., 2012). Roflumilast diminishes histamine-induced microvascular permeability, and reduces leukocyte-endothelial cell interactions in rat mesenteric postcapillary venules. Mechanically, roflumilast inhibits neutrophil adhesion in TNF-α-treated HUVEC, and reduces CD11b expression in N-formyl-methionyl-leucyl-phenylalanine (fMLP)-induced neutrophils (Sanz et al., 2007).

### 5.4 PDE4 inhibitors reduce inflammation in cerebral vascular

Rolipram improves the memory and learning abilities of rats with dementia induced by sodium arsenite-induced cerebral vascular endothelial dysfunction. Additionally, rolipram suppresses the activity of brain acetylcholinesterase, brain oxidative stress, and neutrophil count in dementia rats (Virk et al., 2021). In subarachnoid hemorrhage mice, roflumilast reduces the levels of IL-1β, IL-6, and TNF-α in the brains, decreases the number of apoptotic neurons, and improves neural functional damage and cerebral inflammation (Wu et al., 2017). FCPR16, a novel PDE4 inhibitor, inhibits TNF-α, IL-6 and IL-1β expression, and cell apoptosis via the cAMP/CREB pathway. It improves neural function and reduces cerebral infarction volume in brain ischemia-reperfusion injury rats, with lower emetic potential (Chen et al., 2018). In addition, a novel PDE4 inhibitor α-mangostin derivatives have the potential to treat vascular dementia and do not cause emesis to beagle dogs (Liang et al., 2020).

**FIGURE 1:**
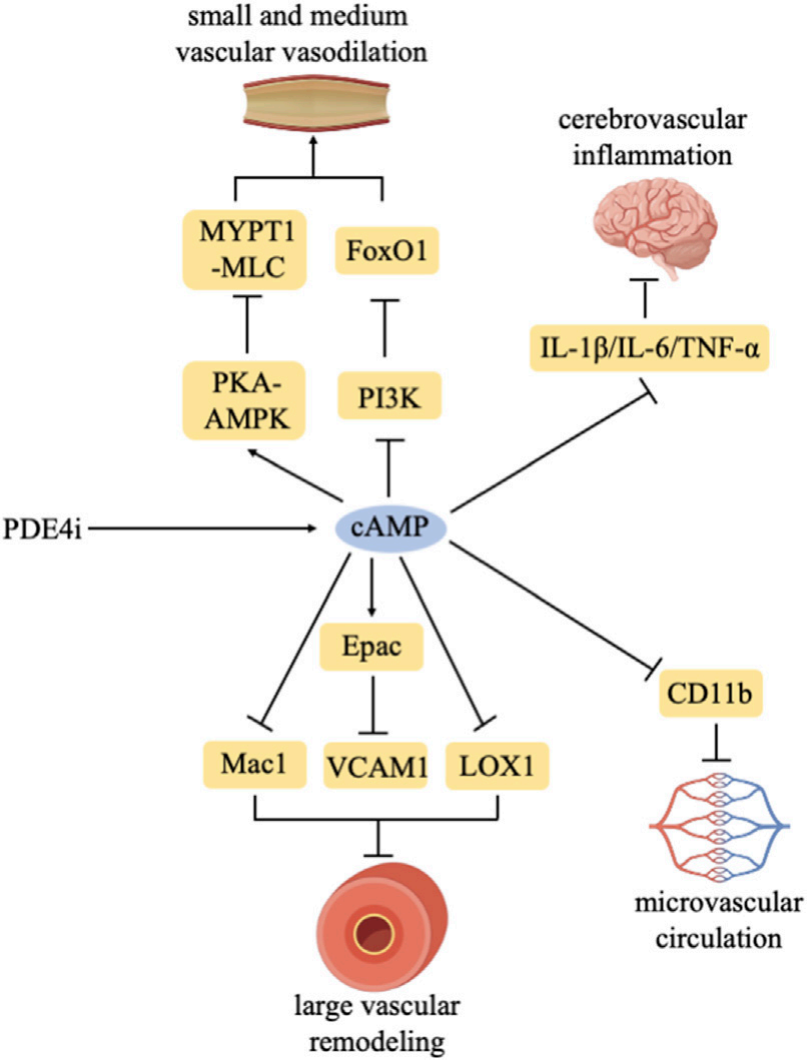
Effects of PDE4 inhibitors on vascular dysfunction.

In conclusion, PDE4 inhibitors play a crucial role in conditions stemming from various vascular dysfunctions and exhibit a certain alleviative effect (Figure 1).

PDE4 inhibitors increase intracellular cAMP levels. PDE4 inhibitors delay large vascular remodeling by regulating Mac1/Epac-VCAM1/LOX1. PDE4 inhibitors promote the vasodilation of small and medium vascular through the PKA-AMPK/MYPT1-MLC/PI3K-FoxO1 pathway. PDE4 inhibitors improve microvascular circulation by inhibiting CD11b. PDE4 inhibitors inhibit cerebrovascular inflammation by reducing IL-1β/IL-6/TNF-α expression.

## 6 Discussion

Currently, roflumilast, apremilast, and crisaborole have been sequentially approved for treating inflammatory airway and skin diseases. Additionally, an increasing number of clinical trials are exploring the potential applications of PDE4 inhibitors for other indications, thereby expanding their clinical use. Recently, the topical roflumilast has been studied in several skin diseases. In clinical trial phase 3, 457 patients with seborrheic dermatitis are randomly assigned to roflumilast foam 0.3% (*n* = 304) or vehicle (*n* = 153) for 8 weeks 79.5% of roflumilast-treated and 58.0% of vehicle-treated patients meet the primary endpoint (*p* < 0.001) (Blauvelt et al., 2024). Furthermore, 39 patients with Behcet’s syndrome are treated with apremilast (*n* = 19) and placebo (*n* = 20) for 12 weeks in a clinical trial. 57.9% patients in the apremilast group achieves complete resolution of oral ulcers, compared to 25.0% in the placebo group. Clinical benefits were sustained over 28 weeks of continued apremilast treatment (Takeno et al., 2022). In a recent clinical trial, the efficacy and safety of crisaborole in treating stasis dermatitis (SD) are evaluated. 65 patients receive crisaborole or vehicle (1:1) for 6 weeks. Central reader (dermatologist) photograph assessments show significant improvement (−52.5% vs. −10.3%, *p* = .0004). Crisaborole effectively ameliorates the signs and symptoms of SD (Silverberg et al., 2024).

Despite the increasing number of clinical trials on PDE4 inhibitors, their clinical use is largely hindered by adverse reactions such as nausea, vomiting, and gastrointestinal reactions. Future research should aim to reduce these side effects while maintaining or enhancing the drugs’ efficacy. To minimize the adverse reactions of PDE4 inhibitors, the following three improvement strategies are currently under consideration.

### 6.1 Design effective isoform-specific inhibitors or allosteric modulators

Study indicates that PDE4D regulates the activity of α2-adrenergic receptors and is the isoform most associated with side effects like vomiting (Schepers et al., 2023). Therefore, isoform-specific PDE4 inhibitors may offer more effective therapeutic approaches (Paes et al., 2021). Phenylalanine is located at position 196 of the upstream conserved region 2 (UCR2) in PDE4D, whereas tyrosine is found at position 274 in PDE4A, PDE4B, and PDE4C. Allosteric modulators targeting UCR2 reduce interaction with the active site, partially inhibiting cAMP hydrolysis. This results in a maximum inhibition rate exceeding 50%, potentially reducing side effects like vomiting in humans and animals (Gurney et al., 2011).

A clinical trial from New England in 2022 reported the efficacy and safety of PDE4B inhibitor (BI 1015550) in patients with idiopathic pulmonary fibrosis (IPF). 147 IPF patients underwent 12 weeks of treatment with BI 1015550 (*n* = 98) or placebo (*n* = 49). The results show that the median change in forced vital capacity (FVC) in the BI 1015550 group is an increase of 5.7 mL, while it decreases by 81.7 mL in the placebo group. This indicates that BI 1015550 is superior to placebo. Although the overall safety of BI 1015550 is acceptable, diarrhea remains the most common adverse reaction. And 13 patients discontinues treatment due to adverse events. PDE4B inhibitors BI 1015550 have anti-fibrotic effects, preventing the decline in lung function in IPF patients, but still have gastrointestinal side effects.

Although the active sites of various PDE4 isoforms share significant similarities, this remains a major challenge. However, isoform-specific inhibitors may be discovered in the future based on existing structural information.

### 6.2 Combination therapy

In COPD treatment, roflumilast combining bronchodilators—long-acting β2-adrenergic receptor agonists (LABA) and long-acting antimuscarinic agents (LAMA)—is significantly more effective than monotherapy. In clinical trial phase IV, 2,354 patients with COPD are randomized 1:1 to receive roflumilast or placebo, plus inhaled corticosteroid/LABA fixed-dose combination (FDC), for 52 weeks. Compared to placebo, roflumilast reduces the rate of COPD acute exacerbations by 18%. The combination therapy of roflumilast and FDC improved lung function and health status in COPD patients. In addition, Parikh et al. discovered that combining PDE4 inhibitors with adenylyl cyclase (AC) inhibitors, β-adrenergic receptor agonists, glucocorticoids, calcium channel blockers, oligonucleotides, cytokine inhibitors, nitric oxide synthase inhibitors, and cyclooxygenase-2 (COX-2) inhibitors is effective in treating inflammatory airway diseases. This combination therapy shows efficacy comparable to LABA and LAMA combination therapy (Parikh and Chakraborti, 2016).

### 6.3 Altering the route of administration

GSK256066 is a highly specific inhalable PDE4B inhibitor. Studies have shown that rats treated with GSK256066 experience milder gastrointestinal adverse reactions (Tralau-Stewart et al., 2011). Moreover, in clinical trial phase IIa, 104 patients with COPD are randomized. GSK256066 increases post-bronchodilator Forced Expiratory Volume 1 (FEV1). There are no serious adverse events in patients receiving GSK256066. The overall incidence of gastrointestinal adverse events was low in all treatment groups. Thus, it is utilized for inhalation therapy in the treatment of inflammatory airway diseases. Furthermore, crisaborole is topically applied to treat skin inflammation. Clinical trials have demonstrated that unlike systemic treatment, the local application of 2% crisaborole ointment significantly improves safety and does not cause notable gastrointestinal adverse reactions (Paller et al., 2016).

## 7 Conclusion

In summary, PDE4 inhibitors represent a significant advancement in the treatment of inflammatory diseases, particularly in pulmonary and skin diseases. They increase cAMP levels and regulate cellular processes involved in inflammation, making them particularly suitable for treating diseases characterized by vascular dysfunction. Studies indicates that PDE4 inhibitors inhibits large vascular remodeling, promote small and medium vascular vasodilation, improve microcirculation, and alleviate inflammation in cerebral vascular. Increasing evidence suggests the potential of PDE4 inhibitors in alleviating vascular inflammation and remodeling, implying their broader therapeutic significance. However, the precise mechanisms by which PDE4 inhibitors act in vascular diseases remain unclear. Although roflumilast, apremilast, and crisaborole have achieved considerable success, their clinical application is still limited by gastrointestinal adverse reactions. Designing isoform-specific inhibitors, exploring combination therapies, and altering routes of administration provide promising avenues for reducing adverse reactions to PDE4 inhibitors. Although these strategies partially address the limitations of current therapies, maximizing or retaining the efficacy of PDE4 inhibitors while minimizing adverse reactions remains an urgent issue. Future research efforts should focus on improving the safety and efficacy of PDE4 inhibitors, elucidating their mechanisms of action in vascular diseases, expanding their clinical utility, and providing new treatment strategies for inflammation and vascular diseases.
